# Association between Soy Food and Dietary Soy Isoflavone Intake and the Risk of Cardiovascular Disease in Women: A Prospective Cohort Study in Korea

**DOI:** 10.3390/nu13051407

**Published:** 2021-04-22

**Authors:** Jihyun Im, Kyong Park

**Affiliations:** Department of Food and Nutrition, Yeungnam University, Gyeongsan 38541, Korea; jhim@ynu.ac.kr

**Keywords:** women, soy food, soy isoflavone, cardiovascular diseases, cohort study

## Abstract

The association between soy food and soy isoflavone intake and cardiovascular disease (CVD) risk is uncertain, especially in women. We aimed to investigate this association in Korean women. We analyzed data from the Korean Genome and Epidemiology Study, including 4713 Korean women aged 40–69 years with no CVD or cancer at baseline. Dietary information was obtained using a validated semi-quantitative food frequency questionnaire, and the incidence of CVD was assessed using biennial self-reported questionnaires on medical history. The mean follow-up time was 7.4 years, during which 82 premenopausal and 200 postmenopausal women reported CVD incidence. The highest tofu, total soy foods, and dietary soy isoflavone intake groups were significantly associated with a decreased CVD risk in premenopausal women (tofu: hazard ratio (HR) 0.39; 95% confidence interval (CI), 0.19–0.80; total soy food: HR 0.36; 95% CI, 0.18–0.70; dietary soy isoflavones: HR 0.44; 95% CI, 0.22–0.89), whereas no association was observed in postmenopausal women. Other soy foods showed no association with CVD incidence. Dietary soy isoflavones and total soy foods are associated with a decreased CVD risk in premenopausal women. Among soy foods, only tofu showed significant health benefits.

## 1. Introduction

Cardiovascular diseases (CVDs), major causes of premature deaths and chronic disabilities, are responsible for a heavy disease burden globally [1,2]. The prevalence of CVD may differ on the basis of income levels and regional characteristics [3], and the high prevalence of CVD in South Korea, with its rapidly aging population, is a pressing public concern [4,5]. According to the 2019 cause of death statistics published by Statistic Korea, 117.4 deaths per 100,000 persons were from CVD [4], and the disease burden of CVD (i.e., stroke) in Korea is higher than the average of that in countries within the Organization for Economic Cooperation and Development [6]. In addition, CVD is commonly included in the top five causes of death for both men and women; however, CVD mortalities were higher in women than in men [4].

The risk of CVD can be reduced with multifaceted lifestyle changes, including dietary changes [7,8,9]. In terms of dietary changes, the association between the dietary intake of soy foods (soybeans) and CVD risk has been frequently investigated [10,11,12]. A recent meta-analysis found that a higher intake of soy foods is associated with a lower CVD risk, especially in women [10]. Isoflavones, a class of phytoestrogens, are the functional component of soybeans, with antioxidant properties that help prevent chronic diseases, especially in middle-aged women [13]. Previous studies have demonstrated that the role of isoflavone intake in preventing CVD differs on the basis of menopausal status [14,15]. For example, Nurses’ Health Study and Nurses’ Health Study II showed that isoflavones are beneficial in reducing CVD risk in women before menopause (hazard ratio [HR] 0.64; 95% confidence interval [CI] 0.45–0.93) [14]. This suggests that the estrogen status may affect the association between isoflavone intake and CVD risk [15].

According to the Korea National Health and Nutrition Examination Survey (KNHANES), Koreans consume isoflavones from various soy foods [16], and it has been confirmed that women, particularly middle-aged women, consumed soy foods more frequently [17]. Nevertheless, very few studies have investigated the association between the soy food/soy isoflavone intake and CVD risk in Korean women, and to the best of our knowledge, no prospective cohort studies have been conducted so far. In this study, using data from the Ansan–Ansung cohort [18], we aimed to prospectively analyze the association between soy food/soy isoflavone intake and CVD risk among women.

## 2. Materials and Methods

### 2.1. Study Population

The Ansan–Ansung cohort is a population-based study cohort comprising a part of the Korean Genome and Epidemiology Study, which was conducted to investigate frequently observed chronic diseases and various genetic and epidemiological risk factors in the Korean population. A detailed description of the cohort, along with a flow diagram of the baseline recruitment and follow-up processes, including the reasons for loss to follow-up, has been published previously [18]. Briefly, a baseline survey for the Ansan–Ansung cohort was conducted between 2001 and 2002, with 10,030 registered participants aged 40–69 years, residing in Ansan and Ansung in Gyeonggi Province, South Korea. In the urban area of Ansan, 5012 participants (response rate, 45.7%) were recruited using a random sampling method based on local telephone directory information, whereas 5018 (response rate, 69.6%) were recruited using a cluster sampling method based on randomly selected administrative regions in the rural areas of Ansung. The general distribution characteristics of participants recruited across both regions were similar to those of participants who had not been recruited. We used data from baseline (2001–2002) to fifth follow-up (2011–2012), in which participants were followed for 10 years (Supplementary Materials Figure S1).

Detailed information, including demographic characteristics, lifestyle habits (physical activities, smoking status, and alcohol consumption), dietary habits, and medical records of the participants, was collected using a structured questionnaire in an interview.

After excluding 498 participants with a history of CVD or cancer at baseline (those who responded positively to one or more of the questions concerning CVD- or cancer-related diagnoses, treatment, or medication use), 390 participants with a daily energy intake of <500 kcal or >5000 kcal [19], 66 participants with missing data on soy food intake, and 4363 men, our analysis comprised 2771 premenopausal and 1942 postmenopausal women (Figure S2). Written informed consent was obtained from all participants. Data collection and analysis for this study were approved by the Institutional Review Boards (IRBs) of the Korea Centers for Disease Control and Prevention and Yeungnam University (IRB numbers: KU-IRB-15-EX-256-A-1 and 7002016-E-2016-003, respectively).

### 2.2. Dietary Assessment

Dietary assessment was conducted during the baseline survey (2001–2002) and again during the second follow-up survey (2005–2006). A trained investigator collected the dietary information of the participants during the interview using the validated semi-quantitative food frequency questionnaire (SQFFQ) [20]. The calculated average servings of food intake have been described elsewhere [21,22]. Briefly, the intake frequency (from ‘almost never’ to ‘3 times daily’ on a 9-point scale) and intake amount (from ‘small’ to ‘large’ on a 3-point scale) for food items were obtained, and the amount of food intake per week was calculated using the intake frequency per week and corresponding serving size. We used the mean of the amount of food intake per week obtained from the baseline and second follow-up surveys to minimize the potential misclassification of dietary information. Among the 4713 participants who completed the baseline SQFFQ and met the inclusion criteria of this study, 1208 participants had missing dietary data at the second follow-up; therefore, we imputed these missing data using the fully conditional specification approach [23]. For the sum of intake of separate foods with different moisture contents, we used the KNHANES conversion factor to convert the dry weights to wet weights [16]. There were four soy foods listed on the SQFFQ, and their corresponding serving sizes were as follows: (1) legumes (soybeans and peas; 12 g per serving) and bean sprouts (soybean sprouts and mungbean sprouts; 40 g per serving); (2) tofu (regular tofu, soft tofu, and extra soft tofu; 60 g per serving); (3) fermented soy paste (Doenjang, Cheonggukjang, and Ssamjang; 9 g per serving); and (4) soymilk (200 g per serving).

### 2.3. Estimation of Soy Isoflavone Intake

Data regarding soy isoflavone intake were obtained from the Food Functional Composition Table published by the Rural Development Administration (RDA) [24], the United States Department of Agriculture (USDA) Database for the Isoflavone Content of Selected Foods Release 2.1 [25], and Phenol-Explorer 3.0 [26]. In cases of duplicated data from multiple resources, the RDA Food Functional Composition Table was used as the primary resource. Furthermore, in cases where no domestic data were available, the data values from the USDA Database for the Isoflavone Content of Selected Foods Release 2.1 and Phenol-Explorer 3.0 were used. We additionally used standard recipes from the Computer-Aided Nutritional Analysis Program 5.0 to calculate the amount of soy in meals. The food items included in the calculation of the total soy isoflavone content comprised soy foods (soybeans [including soybean sprouts], tofu, fermented soy paste, and soymilk). The daily intake of soy isoflavones (mg/day) was calculated using servings per week, intake amount per serving (g/serving), and isoflavone content per 100 g for each food item. The intake of isoflavone supplements was not assessed during the baseline and follow-up surveys.

### 2.4. Incidence of CVD Events during the Study Period

The incidence of CVD was determined at the biennial follow-up assessment, during which a trained investigator individually interviewed the participants using a structured survey and confirmed the occurrence of CVD. The incidence of CVD was defined as being newly diagnosed with CVD or being prescribed CVD-related medications by a physician. CVD cases included cases of myocardial infarction, coronary artery disease, congestive heart failure, or stroke; and resulting in coronary bypass surgery, coronary angioplasty, or coronary stent insertion. The Ansan–Ansung study staff contacted participants who did not attend the follow-up interview and survey via telephone or door-to-door visits [27]. On the basis of a previous report, there was 93% concordance between the medical records and self-reported CVD in this cohort [28].

### 2.5. General Characteristics and Health Information of the Participants

Age, sex, monthly household income, residential area, smoking status, alcohol consumption, and physical activity were assessed using the self-reported questionnaire from the baseline survey. The monthly household income of the participants was categorized into quartiles, whereas their physical activity levels were assessed using metabolic equivalents (h/week) calculated using duration and weighting factors, on the basis of the intensity of their activity (walking, moderate exercise, or intense physical activity) [29]. Health check-up surveys were conducted at the Ajou University Community Health Center, Ansung, and the Institute of Human Genomics at Korea University Ansan Hospital. In addition, anthropometric information (height [m] and weight [kg]) was collected, and fasting blood tests were performed by a trained expert using standardized protocols [30]. Body mass index (BMI, kg/m^2^) was calculated by dividing the value of the measured weight by the square of height. Blood pressure was measured at least twice in a seated position using a mercury sphygmomanometer (Baumanometer, W.A. Baum Co., Inc., Copiague, NY, USA), and the mean value of multiple systolic/diastolic pressure measurements was used in this study. Venous blood was collected using a Vacutainer needle (22–23 gauge) after fasting for at least 8 h, and the collected blood was stored in an 8.5 mL serum separator tube with a 2-dimensional barcode, a 10 mL EDTA tube, and a 3 mL EDTA tube at 4 °C. Blood collected for physicochemical examination was transferred to Seoul Clinical Laboratories (Seoul, Republic of Korea) on the day of collection for further analysis [31]. The fasting blood glucose, total cholesterol (TC), triglyceride (TG), and high-density lipoprotein–cholesterol (HDL–C) levels were evaluated using enzymatic methods (Adivia 1650, Siemens, Tarrytown, NY, USA). The low-density lipoprotein-cholesterol (LDL-C) level was calculated from the TC, HDL-C, and TG levels [32].

### 2.6. Statistical Analysis

After classifying the participants according to their menopausal status, they were divided into quartiles on the basis of their dietary intake levels of total soy foods and soy isoflavones. Categorical and continuous variables were analyzed using the χ^2^ test and generalized linear regression model, respectively. Potential confounding factors were selected on the basis of a preliminary analysis and literature search [33,34]. Based on this information, we evaluated three covariate models using the Cox proportional hazard model. Model 1 was adjusted for age, whereas model 2 was adjusted for age along with residential area, monthly household income, smoking status, alcohol consumption, physical activity, and BMI. Model 3 included all adjustments from Model 2 as well as intake of meats, fish and seafood, fruits, vegetables, dietary supplements, fat, cholesterol, and history of hypertension, dyslipidemia and diabetes. *P* for trend was calculated using the median value of quartiles. Furthermore, all intake levels were energy-adjusted using the residual method [35]. The interactions between key exposure variables and demographical characteristics, lifestyle factors, and dietary factors were evaluated using multiplicative terms, and menopause status was considered to be a potential effect modifier. All statistical analyses were performed using Statistical Analysis System version 9.4 (SAS Institute, Cary, NC, USA). All statistical tests were two-sided; *p*-values < 0.05 were considered significant.

## 3. Results

### 3.1. General Characteristics of the Participants

Over a mean follow-up period of 7.4 years, 82 and 200 cases of CVD were confirmed in pre- and postmenopausal women participants, respectively. The general characteristics of the participants were compared after dividing them according to menopausal status, and they were categorized into quartiles on the basis of their total soy food intake levels (Table 1). For premenopausal women, a higher total soy food intake level was positively associated with age (*p* < 0.001), BMI (*p* < 0.001), systolic and diastolic blood pressure (*p* < 0.001), TG (*p* = 0.005), TC (*p* < 0.001), LDL–C (*p* = 0.02), fasting blood glucose (*p* = 0.045), and history of hypertension (*p* < 0.001). Furthermore, higher intake of total soy foods was associated with increased consumption of vegetables (*p* < 0.001) and fish and seafood (*p* < 0.001). Interestingly, postmenopausal women exhibited distinct trends. Although the majority of general characteristics, lifestyle habits, and biochemical characteristics in postmenopausal women were similar to those in their premenopausal counterparts, a higher proportion of non-drinkers (*p* = 0.01) and participants with a history of diabetes (*p* = 0.03) showed increased total soy food intake among postmenopausal women. Moreover, increased consumption of vegetables (*p* < 0.001) and fish and seafood (*p* = 0.001) was also observed among postmenopausal women with a higher intake of total soy foods.

### 3.2. Food Groups Contributing to Soy Isoflavone Intake

The food groups that contributed to soy isoflavone intake are shown in Table 2. The soy isoflavone intake levels in pre- and postmenopausal women were 15.87 mg/day (standard deviation [SD], 11.37 mg/day) and 15.22 mg/day (SD, 11.94 mg/day), respectively. Interestingly, the food groups that contributed highly to soy isoflavone intake were identical for both pre- and postmenopausal women, among which soybeans and soybean sprouts contributed the most to soy isoflavone intake (premenopausal, 35.45%; postmenopausal, 33.23%), followed by tofu, fermented soy paste, and soymilk.

### 3.3. Total Soy Food/Soy Isoflavone Intake and the Incidence of CVD

Table 3 shows the HRs and 95% CIs of CVD incidence according to the total soy food and soy isoflavone intake levels. All statistical models showed no significant association between the intake of total soy foods and soy isoflavones and CVD incidence in postmenopausal women. However, in premenopausal women, increased total soy food and soy isoflavone intake was significantly associated with a decreased CVD risk. In Models 1 and 2, there were inverse associations between total soy food and soy isoflavone intake and CVD risk, although the linear trends were not statistically significant. In the fully adjusted Model 3, total soy food intake was significantly inversely associated with CVD risk (highest vs. lowest: HR, 0.36; 95% CI, 0.18–0.70; *p* for trend = 0.01). In addition, increased soy isoflavone intake was significantly associated with decreased CVD risk (highest vs. lowest: HR, 0.44; 95% CI, 0.22–0.89; *p* for trend = 0.03).

### 3.4. Association between Individual Soy Food Intake and CVD Incidence

Table 4 shows the HRs and 95% CIs of CVD incidence according to the intake level of individual soy foods stratified by menopausal status. In Model 3, a significant association was observed between increased tofu intake and decreased CVD risk in premenopausal women (highest vs. lowest: HR, 0.39; 95% CI, 0.19–0.80 *p* for trend = 0.01), which was not observed with other soy foods (highest vs. lowest: soybeans HR, 0.84; 95% CI, 0.43–1.63; *p* for trend = 0.60; soymilk HR, 1.02; 95% CI, 0.43–2.46; *p* for trend = 0.66). For fermented soy paste, a significant association was evident for the third quartile (Model 3: HR, 0.40; 95% CI, 0.19–0.86) compared with the lowest quartile, but a linear trend was not observed (*p* for trend = 0.29). In contrast, among postmenopausal women, none of the statistical models showed any significant association between intake of individual soy food and incidence of CVD.

## 4. Discussion

In the current study, we found that higher total soy food and soy isoflavone intake was significantly associated with a decreased CVD risk in premenopausal women; however, these associations were not observed in postmenopausal women.

Soy foods are an excellent source of essential nutrients, including proteins, soluble dietary fibers, unsaturated fatty acids, iron, and isoflavones, that constitute bioactive components [13], which have been reported to prevent CVD through several independent functions [36]. First, soy proteins reduce oxidative stress and improve endothelial function to reduce LDL–C levels. Moreover, the soluble dietary fibers induce the feeling of satiety, which prevents excessive eating and therefore aids in weight loss [13]. Second, isoflavones exhibit antioxidant properties, which protect blood vessels from oxidative stress-induced damage, and vasodilatory properties, which promote the release of prostaglandin and anti-inflammatory molecules [37]. Third, isoflavones can act as estrogen agonists in women with low blood estrogen concentration levels through binding to estrogen receptor-β and as estrogen antagonists in women with high blood estrogen concentration levels [38]. In other words, isoflavones, also referred to as phytoestrogens, selectively bind to the estrogen receptor and exert effects similar to that of estrogen [38] by protecting blood vessels through antioxidant characteristics and improving cardiac function through binding to estrogen receptors in the cardiac cells [39]. Therefore, soy isoflavones have shown beneficial effects on the treatment and prevention of cardiovascular disease, cholesterol lowering, and menopausal symptoms as a nutraceutical [40,41].

Associations between the level of isoflavone intake and CVD risk in pre- and postmenopausal women have been different in the three studies conducted in Japan [42] and the United States of America [14,43]. A study on a Japan Public Health Center-based cohort revealed that isoflavone intake was inversely associated with CVD risk in postmenopausal women (HR 0.25; 95% CI, 0.14–0.45), whereas no such benefit was observed in premenopausal women (HR 0.62; 95% CI, 0.23–1.70) [42]. However, in the Iowa Women’s Health Study conducted among 34,489 postmenopausal women, the intake of isoflavone was not associated with CVD mortality (relative risk 1.05; 95% CI, 0.91–1.21) [43]. Moreover, the most recent study using the Nurses’ Health Study and Nurses’ Health Study II reported that isoflavone intake was negatively associated with coronary heart disease only in premenopausal women [14]. The HR (95% CI) comparing participants with the highest and lowest intakes of isoflavone were 0.64 (0.45–0.93; *p* for trend = 0.01) among premenopausal women, with no such significant association observed among postmenopausal women [14]. Our results are in line with those of the studies from the USA, and we speculate that the different health effects of soy foods and soy isoflavones on the risk of CVD may be due to an interrelation between circulating estrogen and endothelial estrogen receptor expression levels [15]. Notably, postmenopausal women tend to lack circulating estrogen levels, resulting in a decreased expression of endothelial estrogen receptors, which is regulated by circulating estrogen [15]. In addition, estrogen can act as an antioxidant by removing free radicals through the phenol hydroxylase pathway and exert cardioprotective functions [39]. The CVD-reducing effect of isoflavones may be attributed to a complex interaction between isoflavone intake, estrogen, and estrogen receptor expression levels; therefore, we assume that the synergic effects of dietary soy isoflavones (as phytoestrogens or antioxidants) could be observed better in premenopausal women. Nevertheless, more biological and mechanistic studies are needed to elucidate the complex relationships between menopause, soy isoflavone, and CVD risk.

In our study, tofu consumption was found to significantly reduce CVD risk. Tofu, which is made from soybeans, is one of the most frequently consumed food items in Korea. The complex interplay between isoflavones and other nutrients, such as vegetable proteins, dietary fibers, and calcium in tofu products, has been reported to be positively associated with a reduction in CVD risk [13]. In our study, premenopausal women had a slightly higher intake of tofu and soy isoflavones than did postmenopausal women. On average, pre- and postmenopausal women consumed 4.80 and 4.42 servings of tofu per week and 15.87 and 15.22 mg of dietary soy isoflavones per day, respectively.

Despite the several interesting findings, this study has some limitations. First, although we attempted to adjust for all potential confounding factors using multivariate models, there may have been residual confounding factors that were not accounted for because of the observational nature of the study. Second, the isoflavone supplement intake was not thoroughly analyzed in this study. Third, our findings cannot be generalized to other populations, especially those with variable ranges of soy food and soy isoflavone intake. Fourth, our study population was relatively healthy and had lower risk of CVD at baseline because East Asian populations tend to have lower coronary artery disease mortality and incidence than Western populations [44]. Thus, the small number of CVD cases during the follow-up may led to low statistical power to detect any association between soy isoflavone intake and CVD risk and thus false negative error potential (type 2 error). In addition, CVD was determined using self-reported questionnaires, and missing values of the follow-up dietary information was imputed. Moreover, SQFFQ requires good participant memory; thus, there may be non-differential misclassification errors, which may produce bias toward the null. However, we tried to minimize misclassification bias using a validated SQFFQ and to minimize measurement errors using the mean value of dietary data collected over two assessments. To the best of our knowledge, this study is the first prospective study to assess the association between soy food/soy isoflavone intake and CVD risk in Korean women. In the future, data from this study may be used to establish dietary guidelines for soy food and soy isoflavone intake in Korea.

## 5. Conclusions

In this prospective investigation of the association between soy food/soy isoflavone intake and CVD risk in a representative Ansan–Ansung cohort, premenopausal women with the highest tofu, total soy food, and soy isoflavone intake exhibited a significantly reduced CVD risk, whereas there was no such benefit observed in postmenopausal women. Future large-scale, randomized clinical trials are warranted to identify the causal relationship between soy isoflavone intake and CVD and to determine the adequate amount of soy isoflavone intake necessary for the prevention of CVD.

## Figures and Tables

**Table 1:** Baseline characteristics of the study population according to the quartiles of energy-adjusted total soy food intake.

	Quartiles of Energy-Adjusted Total Soy Food Intake				*p* ^1^
	1 (Low)	2	3	4 (High)	
Premenopause					
No. of participants	692	693	693	693	
Age, year	47.25 ± 7.43	47.57 ± 7.56	47.81 ± 7.56	50.63 ± 8.73	<0.001
Monthly household income, n (%)					<0.001
Low	208 (30.50)	181 (26.66)	168 (24.63)	253 (37.21)	
Mid-low	119 (17.45)	102 (15.02)	93 (13.64)	119 (17.50)	
Mid-high	255 (37.39)	257 (37.85)	276 (40.47)	211 (31.03)	
High	100 (14.66)	139 (20.47)	145 (21.26)	97 (14.26)	
Residential area, n (%)					<0.001
Ansung	305 (44.08)	236 (34.05)	241 (34.78)	322 (46.46)	
Ansan	387 (55.92)	457 (65.95)	452 (65.22)	371 (53.54)	
Current smokers: yes, n (%)	22 (3.18)	30 (4.33)	25 (3.61)	22 (3.17)	0.62
Current drinkers: yes, n (%)	205 (29.62)	210 (30.30)	216 (31.17)	187 (26.98)	0.35
Physical activity level, n (%)					0.37
Low	235 (35.34)	216 (32.00)	237 (35.06)	219 (32.30)	
Moderate	205 (30.83)	242 (35.85)	229 (33.88)	221 (32.60)	
High	225 (33.83)	217 (32.15)	210 (31.07)	238 (35.10)	
BMI, kg/m^2^	24.48 ± 3.14	24.63 ± 3.10	24.72 ± 3.31	25.19 ± 3.49	<0.001
Systolic blood pressure, mmHg	114.81 ± 16.98	116.02 ± 18.08	115.64 ± 17.95	120.31 ± 20.15	<0.001
Diastolic blood pressure, mmHg	75.70 ± 10.69	76.58 ± 11.83	76.26 ± 11.45	78.59 ± 11.85	<0.001
TG, mg/dL	134.68 ± 80.96	134.51 ± 76.36	137.29 ± 75.98	145.41 ± 79.82	0.005
TC, mg/dL	184.65 ± 34.19	185.00 ± 33.85	187.93 ± 35.54	190.71 ± 37.67	<0.001
HDL-C, mg/dL	46.12 ± 9.55	46.03 ± 10.16	46.56 ± 10.01	46.44 ± 10.68	0.42
LDL-C, mg/dL	111.60 ± 30.87	112.07 ± 29.84	113.92 ± 31.75	115.19 ± 31.69	0.02
Fasting blood glucose, mg/dL	83.49 ± 19.51	84.94 ± 23.11	84.99 ± 20.07	85.95 ± 21.66	0.045
Hypertension, %	59 (8.53)	73 (10.53)	76 (10.97)	124 (17.89)	<0.001
Dyslipidemia, %	12 (1.73)	11 (1.59)	16 (2.31)	11 (1.59)	0.71
Diabetes, %	27 (3.90)	27 (3.90)	33 (4.76)	39 (5.63)	0.35
Dietary supplements, %	124 (17.92)	142 (20.49)	157 (22.66)	161 (23.23)	0.06
Total energy intake, kcal/day	1976.22 ± 577.03	1739.74 ± 495.20	1751.76 ± 479.66	1892.13 ± 618.76	0.27
Vegetables intake, g/week ^1^	1171.86 ± 481.82	1325.31 ± 494.50	1425.34 ± 580.45	1572.86 ± 646.93	<0.001
Fruits intake, g/week ^1^	612.74 ± 525.53	689.02 ± 545.22	742.99 ± 678.75	632.23 ± 665.14	0.78
Meat intake, g/week ^1^	329.69 ± 375.91	424.27 ± 494.51	427.33 ± 468.27	331.00 ± 402.24	0.38
Fish and seafood intake, g/week ^1^	235.06 ± 170.96	289.49 ± 191.55	328.24 ± 230.88	316.26 ± 271.73	<0.001
Postmenopause					
No. of participants	485	486	486	485	
Age, year	57.80 ± 6.96	58.49 ± 6.80	58.32 ± 6.73	59.71 ± 6.34	<0.001
Monthly household income, n (%)					0.001
Low	161 (33.89)	164 (34.67)	183 (38.53)	209 (43.91)	
Mid-low	117 (24.63)	93 (19.66)	102 (21.47)	91 (19.12)	
Mid-high	68 (14.32)	75 (15.86)	67 (14.11)	86 (18.07)	
High	129 (27.16)	141 (29.81)	123 (25.89)	90 (18.91)	
Residential area, n (%)					<0.001
Ansung	341 (70.31)	299 (61.52)	292 (60.08)	393 (81.03)	
Ansan	144 (29.69)	187 (38.48)	194 (39.92)	92 (18.97)	
Current smokers: yes, n (%)	21 (4.33)	20 (4.12)	14 (2.88)	20 (4.12)	0.63
Current drinkers: yes, n (%)	118 (24.33)	101 (20.78)	90 (18.52)	77 (15.88)	0.01
Physical activity level, n (%)					0.004
Low	160 (33.97)	176 (36.90)	161 (34.04)	133 (28.00)	
Moderate	148 (31.42)	166 (34.80)	169 (35.73)	152 (32.00)	
High	163 (34.61)	135 (28.30)	143 (30.23)	190 (40.00)	
BMI, kg/m^2^	25.06 ± 3.16	25.18 ± 3.39	25.17 ± 3.34	24.98 ± 3.37	0.57
Systolic blood pressure, mmHg	125.33 ± 19.21	125.40 ± 20.05	126.64 ± 19.56	129.22 ± 19.93	<0.001
Diastolic blood pressure, mmHg	81.72 ± 11.66	80.91 ± 11.28	81.33 ± 11.30	82.58 ± 11.43	0.10
TG, mg/dL	161.45 ± 89.58	158.12 ± 106.22	165.31 ± 89.83	165.39 ± 107.60	0.36
TC, mg/dL	198.48 ± 34.50	197.37 ± 34.24	196.12 ± 36.07	195.83 ± 36.60	0.23
HDL-C, mg/dL	45.08 ± 9.97	44.77 ± 9.68	44.41 ± 10.23	44.49 ± 9.75	0.36
LDL-C, mg/dL	121.11 ± 33.05	120.98 ± 32.51	118.64 ± 32.56	118.25 ± 33.14	0.12
Fasting blood glucose, mg/dL	84.26 ± 15.12	85.15 ± 18.69	85.82 ± 19.82	85.84 ± 16.82	0.18
Hypertension, %	88 (18.14)	117 (24.07)	113 (23.25)	126 (25.98)	0.03
Dyslipidemia, %	9 (1.86)	11 (2.26)	10 (2.06)	8 (1.65)	0.91
Diabetes, %	25 (5.15)	39 (8.02)	43 (8.85)	49 (10.10)	0.03
Dietary supplements, %	101 (20.82)	111 (22.84)	122 (25.10)	125 (25.77)	0.25
Total energy intake, kcal/day	1782.49 ± 499.41	1638.97 ± 438.58	1683.29 ± 424.59	1795.17 ± 519.86	0.06
Vegetables intake, g/week ^1^	1217.97 ± 484.44	1276.71 ± 453.68	1421.17 ± 562.57	1535.43 ± 596.31	<0.001
Fruits intake, g/week ^1^	751.42 ± 558.84	842.10 ± 664.24	903.31 ± 702.63	771.38 ± 556.35	0.95
Meat intake, g/week ^1^	271.14 ± 259.58	345.69 ± 339.37	312.30 ± 343.33	245.14 ± 319.23	0.01
Fish and seafood intake, g/week ^1^	211.86 ± 188.50	250.59 ± 186.40	263.71 ± 204.18	258.88 ± 213.85	0.001

BMI, body mass index; TG, triglyceride; TC, total cholesterol; HDL- C, high-density lipoprotein-cholesterol; LDL-C, low-density lipoprotein-cholesterol. Values are n (%) or mean ± standard deviation. ^1^ *p* values are derived from χ^2^ test for categorical variables and *p* for trends across quartiles of total soy foods were calculated by using linear regression models for continuous variables.

**Table 2:** Contribution of food groups to dietary soy isoflavones among women.

Food Groups	Contribution (%)	Intake (mg/day) ^1^
Premenopause		
Total soy isoflavones	100	15.87 ± 11.37
Soybeans ^2^	35.45	5.75 ± 5.88
Tofu	34.27	5.30 ± 4.96
Fermented soy paste	22.79	3.09 ± 2.71
Soymilk	7.49	1.73 ± 5.12
Postmenopause		
Total soy isoflavones	100	15.22 ± 11.94
Soybeans ^2^	33.23	5.09 ± 5.95
Tofu	30.27	4.57 ± 4.45
Fermented soy paste	27.66	3.53 ± 2.77
Soymilk	8.84	2.04 ± 5.64

^1^ Mean ± standard deviation. ^2^ Includes soybeans and soybean sprouts.

**Table 3:** Hazard ratios and 95% confidence intervals for cardiovascular disease by quartiles of energy-adjusted intake levels of total soy foods and dietary soy isoflavones.

	Quartiles of Energy-Adjusted Intake Levels of Total Soy Foods and Dietary Soy Isoflavones				*p* for Trend ^1^
	1 (Low)	2	3	4 (High)	
Premenopause					
Total soy foods					
Median, servings/week	4.66	7.67	10.62	16.56	
No. of cases (%)	28 (4.05)	17 (2.45)	20 (2.89)	17 (2.45)	
Person-years	4571.77	4822.76	4751.38	4029.70	
Model 1	1	0.53 (0.29–0.98)	0.62 (0.35–1.11)	0.51 (0.28–0.95)	0.07
Model 2	1	0.49 (0.26–0.94)	0.66 (0.37–1.18)	0.48 (0.26–0.89)	0.05
Model 3	1	0.39 (0.20–0.77)	0.55 (0.30–1.03)	0.36 (0.18–0.70)	0.01
Dietary soy isoflavones					
Median, mg/day	6.76	11.77	17.12	26.94	
No. of cases (%)	24 (3.47)	21 (3.03)	21 (3.03)	16 (2.31)	
Person-years	4344.19	4812.05	4663.43	4355.94	
Model 1	1	0.78 (0.44–1.40)	0.80 (0.45–1.44)	0.65 (0.34–1.22)	0.21
Model 2	1	0.74 (0.40–1.35)	0.75 (0.41–1.37)	0.63 (0.33–1.19)	0.19
Model 3	1	0.64 (0.35–1.19)	0.66 (0.35–1.23)	0.44 (0.22–0.89)	0.03
Postmenopause					
Total soy foods					
Median, servings/week	5.27	8.35	11.31	18.03	
No. of cases (%)	52 (10.72)	45 (9.26)	43 (8.85)	60 (12.37)	
Person-years	4184.00	4270.47	4191.17	4140.85	
Model 1	1	0.83 (0.56–1.24)	0.81 (0.54–1.22)	1.07 (0.74–1.56)	0.48
Model 2	1	0.84 (0.56–1.27)	0.80 (0.53–1.21)	1.02 (0.69–1.50)	0.72
Model 3	1	0.75 (0.50–1.15)	0.74 (0.48–1.13)	0.95 (0.63–1.43)	0.88
Dietary soy isoflavones					
Median, mg/day	6.89	11.99	16.68	27.37	
No. of cases (%)	53 (10.93)	58 (11.93)	39 (8.02)	50 (10.31)	
Person-years	4181.73	4178.23	4270.46	4156.08	
Model 1	1	1.14 (0.79–1.66)	0.76 (0.50–1.16)	0.97 (0.66–1.43)	0.61
Model 2	1	1.19 (0.81–1.75)	0.81 (0.53–1.23)	0.99 (0.67–1.47)	0.67
Model 3	1	1.12 (0.75–1.67)	0.79 (0.51–1.22)	1.02 (0.66–1.56)	0.84

^1^*p* for trend was evaluated by assigning the median values of each quartile of dietary intake to a continuous variable. Model 1: adjusted for age; Model 2: additionally adjusted for residential area, monthly household income, smoking, alcohol consumption, physical activity, and body mass index; Model 3: additionally adjusted for intake of meats, fish and seafood, fruits, vegetables, dietary supplements, fat, cholesterol, and history of hypertension, dyslipidemia and diabetes.

**Table 4:** Hazard ratios and 95% confidence intervals for cardiovascular disease by quartiles of energy-adjusted individual soy food intake.

	Quartiles of Energy-Adjusted Individual Soy Food Intake				*p* for Trend ^1^
	1 (Low)	2	3	4 (High)	
Premenopause					
Soybeans ^2^					
Median, servings/week	1.04	2.20	3.61	7.74	
No. of cases (%)	19 (2.75)	23 (3.32)	18 (2.60)	22 (3.17)	
Person-years	4300.12	4786.44	4824.00	4265.04	
Model 1	1	1.04 (0.57–1.91)	0.83 (0.43–1.58)	0.94 (0.51–1.74)	0.78
Model 2	1	1.07 (0.57–2.02)	0.84 (0.43–1.63)	0.93 (0.49–1.75)	0.72
Model 3	1	0.96 (0.49–1.87)	0.79 (0.39–1.59)	0.84 (0.43–1.63)	0.60
Tofu					
Median, servings/week	0.78	1.72	2.73	4.80	
No. of cases (%)	27 (3.90)	21 (3.03)	21 (3.03)	13 (1.88)	
Person-years	4199.55	4563.71	4934.08	4478.26	
Model 1	1	0.77 (0.44–1.37)	0.77 (0.43–1.37)	0.53 (0.27–1.04)	0.07
Model 2	1	0.86 (0.47–1.57)	0.82 (0.45–1.49)	0.53 (0.27–1.06)	0.07
Model 3	1	0.72 (0.38–1.35)	0.68 (0.37–1.27)	0.39 (0.19–0.80)	0.01
Fermented soy paste					
Median, servings/week	1.04	2.01	3.39	5.77	
No. of cases (%)	19 (2.75)	26 (3.75)	13 (1.88)	24 (3.46)	
Person-years	4482.27	4802.98	4720.74	4169.63	
Model 1	1	1.23 (0.68–2.23)	0.58 (0.28–1.17)	0.98 (0.53–1.80)	0.58
Model 2	1	1.16 (0.63–2.12)	0.53 (0.26–1.10)	0.92 (0.50–1.70)	0.50
Model 3	1	0.96 (0.51–1.81)	0.40 (0.19–0.86)	0.76 (0.39–1.46)	0.29
Soymilk					
Median, servings/week	0.00	0.07	0.20	0.72	
No. of cases (%)	15 (2.07)	21 (3.17)	23 (3.32)	23 (3.32)	
Person-years	4487.41	4485.76	4373.32	4829.12	
Model 1	1	1.32 (0.68–2.57)	1.16 (0.60–2.24)	1.21 (0.63–2.32)	0.84
Model 2	1	1.47 (0.74–2.92)	1.18 (0.60–2.32)	1.30 (0.66–2.55)	0.76
Model 3	1	1.61 (0.64–4.03)	0.98 (0.37–2.59)	1.02 (0.43–2.46)	0.66
Postmenopause					
Soybeans ^2^					
Median, servings/week	1.16	2.37	3.99	9.84	
No. of cases (%)	41 (8.45)	51 (10.49)	56 (11.52)	52 (10.72)	
Person-years	4295.16	4213.20	4120.21	4157.93	
Model 1	1	1.29 (0.85–1.94)	1.51 (1.01–2.26)	1.25 (0.83–1.88)	0.58
Model 2	1	1.37 (0.90–2.10)	1.65 (1.09–2.48)	1.18 (0.77–1.80)	0.95
Model 3	1	1.31 (0.84–2.04)	1.73 (1.13–2.65)	1.13 (0.73–1.75)	0.93
Tofu					
Median, servings/week	0.74	1.53	2.55	4.42	
No. of cases (%)	59 (12.16)	54 (11.11)	47 (9.67)	40 (8.25)	
Person-years	4199.46	4166.44	4230.78	4189.82	
Model 1	1	0.93 (0.64–1.35)	0.85 (0.58–1.25)	0.74 (0.50–1.11)	0.13
Model 2	1	1.09 (0.74–1.59)	1.00 (0.67–1.49)	0.80 (0.53–1.22)	0.22
Model 3	1	1.01 (0.68–1.50)	0.94 (0.62–1.43)	0.83 (0.54–1.28)	0.34
Fermented soy paste					
Median, servings/week	1.34	2.54	3.86	6.33	
No. of cases (%)	47 (9.69)	51 (10.49)	50 (10.29)	52 (10.72)	
Person-years	4179.28	4228.39	4206.77	4172.06	
Model 1	1	1.04 (0.70–1.54)	0.98 (0.66–1.46)	1.00 (0.67–1.48)	0.91
Model 2	1	1.06 (0.70–1.60)	1.00 (0.66–1.50)	0.98 (0.65–1.48)	0.83
Model 3	1	0.99 (0.66–1.51)	0.92 (0.61–1.39)	0.91 (0.60–1.39)	0.62
Soymilk					
Median, servings/week	0.00	0.12	0.24	0.82	
No. of cases (%)	42 (8.66)	49 (10.08)	60 (12.35)	49 (10.10)	
Person-years	4255.38	4233.05	4140.53	4157.53	
Model 1	1	1.11 (0.73–1.67)	1.29 (0.87–1.92)	1.10 (0.73–1.67)	0.88
Model 2	1	1.11 (0.73–1.71)	1.30 (0.87–1.96)	1.06 (0.69–1.63)	0.90
Model 3	1	0.96 (0.56–1.64)	0.83 (0.47–1.49)	0.85 (0.51–1.42)	0.61

^1^*p* for trend was evaluated by assigning the median values of each quartile of dietary intake to a continuous variable. ^2^ Includes soybeans and soybean sprouts. Model 1: adjusted for age; Model 2: additionally adjusted for residential area, monthly household income, smoking, alcohol consumption, physical activity, and body mass index; Model 3: additionally adjusted for intake of meats, fish and seafood, fruits, vegetables, dietary supplements, fat, cholesterol, and history of hypertension, dyslipidemia and diabetes.

## Supplementary Materials

The following are available online at https://www.mdpi.com/article/10.3390/nu13051407/s1, Figure S1: Flow diagram of baseline recruitment and follow up for the Korean Genome and Epidemiology study of Ansan-Ansung cohort. Figure S2. Flow chart of participant selection for the analysis. KoGES, Korean Genome and Epidemiology study.
