# Supplementary material for: Association between Soy Food and Dietary Soy Isoflavone Intake and the Risk of Cardiovascular Disease in Women: A Prospective Cohort Study in Korea
Source: Nutrients. 2021 Apr 22;13(5):1407. doi: 10.3390/nu13051407 (PMC8143453; doi:10.3390/nu13051407)
Supplement: Supplementary file 1 [file nutrients-13-01407-s001.zip › nutrients-1174170-SI.pdf]

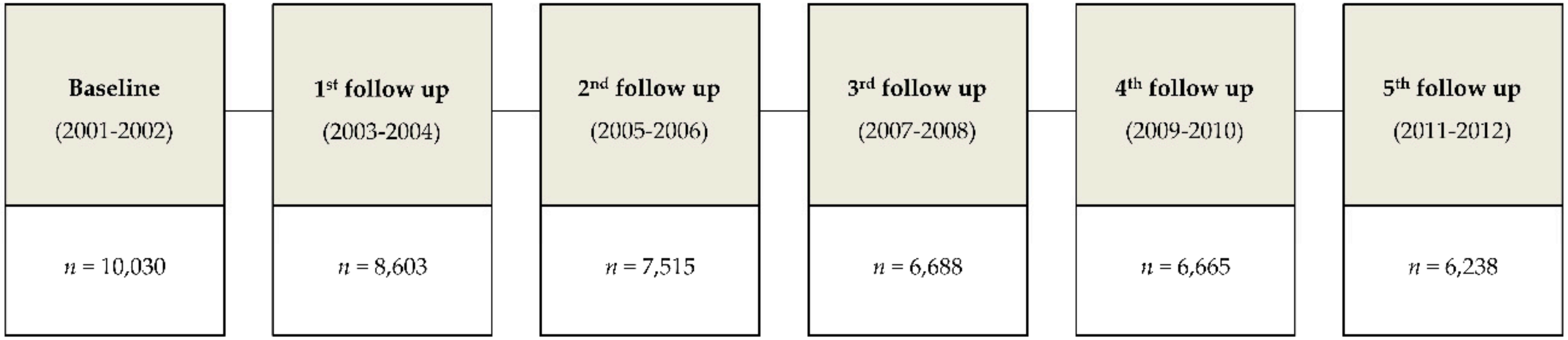

Figure S1. Flow diagram of baseline recruitment and follow up for the Korean Genome and Epidemiology study of Ansan-Ansung cohort.

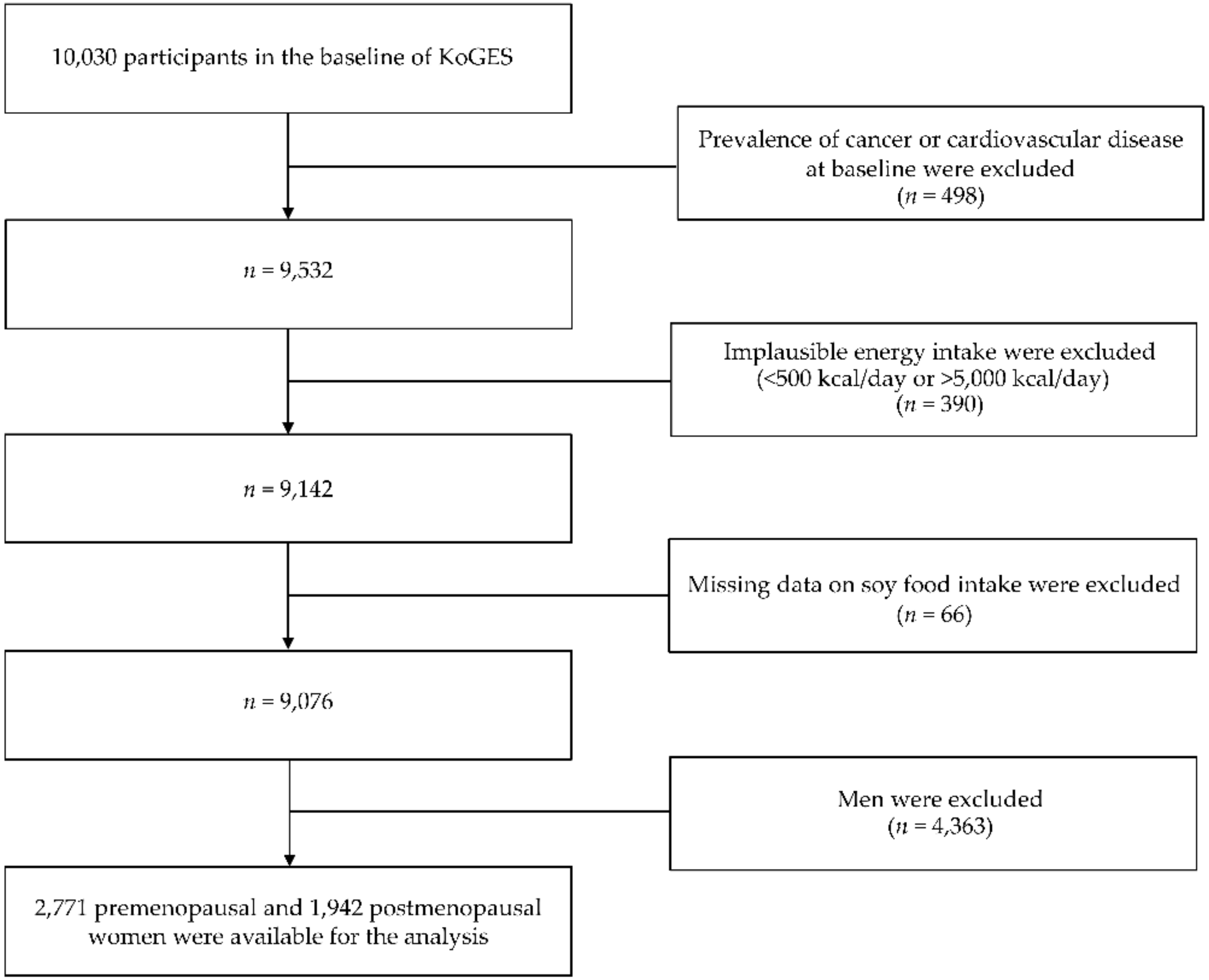

Figure S2. Flow chart of participant selection for the analysis. KoGES, Korean Genome and Epidemiology study.
